# Gaussian Decomposition vs. Semiclassical Quantum Simulation: Obtaining the High-Order Derivatives of a Spectrum in the Case of Photosynthetic Pigment Optical Properties Studying

**DOI:** 10.3390/s23198248

**Published:** 2023-10-05

**Authors:** Andrei P. Razjivin, Vladimir S. Kozlovsky, Aleksandr A. Ashikhmin, Roman Y. Pishchalnikov

**Affiliations:** 1 Belozersky Research Institute of Physico-Chemical Biology, Moscow State University, 119992 Moscow, Russia; wladask@gmail.com; 2 Institute of Basic Biological Problems, Russian Academy of Sciences, 142290 Pushchino, Russia; ashikhminaa@gmail.com; 3 Prokhorov General Physics Institute of the Russian Academy of Sciences, 119991 Moscow, Russia

**Keywords:** chlorophyll, bacteriochlorophyll, carotenoid, gaussian decomposition, absorption spectrum, multimode brownian oscillator model, differential evolution

## Abstract

In this paper, a procedure for obtaining undistorted high derivatives (up to the eighth order) of the optical absorption spectra of biomolecule pigments has been developed. To assess the effectiveness of the procedure, the theoretical spectra of bacteriochlorophyll ***a***, chlorophyll ***a***, spheroidene, and spheroidenone were simulated by fitting the experimental spectra using the differential evolution algorithm. The experimental spectra were also approximated using sets of Gaussians to calculate the model absorption spectra. Theoretical and model spectra can be differentiated without smoothing (high-frequency noise filtering) to obtain high derivatives. Superimposition of the noise track on the model spectra allows us to obtain test spectra similar to the experimental ones. Comparison of the high derivatives of the model spectra with those of the test spectra allows us to find the optimal parameters of the filter, the application of which leads to minimal differences between the high derivatives of the model and test spectra. For all four studied pigments, it was shown that smoothing the experimental spectra with optimal filters makes it possible to obtain the eighth derivatives of the experimental spectra, which were close to the eighth derivatives of their theoretical spectra.

## 1. Introduction

The use of derivatives to increase the spectral resolution was first proposed by Lord Rutherford and was also initially applied in the work of his colleagues [1]. To date, more than a thousand papers have been published in which the term “derivative spectroscopy” is mentioned in the title. The theory and practice of derivative spectroscopy are covered in detail in many books [2,3,4], reviews [5,6,7,8,9,10,11], and studies [7,12,13,14,15,16,17,18,19,20].

Mostly the first or the second derivatives are used, although in the software of modern commercial spectrophotometers it is possible to obtain derivatives of spectra up to four orders. The accuracy of recording spectra is limited by the noise characteristics of the electronics of the spectrophotometers. In modern spectrophotometers, the amplitude of the noise track is usually close to 0.0001 o.u. If, for instance, the long-wavelength absorption peak of chlorophyll ***a*** (Chl) is about one optical unit, then the signal is 10,000 times greater than the noise. However, a single differentiation of the spectrum decreases this ratio to ~100. With a twofold differentiation, the signal will be equal or so to the noise. Therefore, more or less satisfactory spectrum derivatives are obtained only for the first derivative (without smoothing).

In all cases, when it comes to derivatives above the first, different methods of filtering (smoothing) the data are used in order to suppress high-frequency noise. Among the smoothing methods, the most commonly mentioned are adjacent averaging, the Savitsky–Golay algorithm, FFT-filter, and others. Filtering (smoothing) the spectra makes it possible to obtain a higher derivative, but at the same time this derivative turns out to be distorted—the signal bands are broadened, their amplitude drops, and wavy parasitic bands appear. It is easy to show the distortions of the derivatives due to filtering the spectra by modeling, but it is not possible to take into account and correct the distortions. This is especially true for high derivatives, for example, of the eighth order [21,22,23,24]. We have previously used the eighth derivative to reveal the upper exciton band of the bacteriochlorophyll ***a*** (BChl) dimer of the photosynthetic reaction center of purple bacteria [25]. In general, it is difficult to formalize the procedure of filtering (smoothing) and differentiating to obtain high derivatives [26].

If we ignore the formal side of obtaining derivative spectra, we can ask how the spectra of polyatomic dye molecules (pigments, chromophores) and their derivatives should look like, based on physical considerations. Molecular spectra in the near UV, visible, and near IR regions consist of bands corresponding to electronic transitions. In addition to electronic transitions, so-called phonon wings may be present in the spectrum. These bands are due to the interaction of molecular vibrations with the electronic states. In most cases, the contribution of rotational components is too small when pigments are studied in solvents, and their traces are not detected on the spectra.

Ab initio simulations of absorption spectra of polyatomic dye molecules require substantial computational resources, and when it comes to the relatively fast simulation of the obtained spectroscopy data, this type of simulation is not promising. The use of semi-classical quantum theories describing the interaction of the electric field with matter can be considered promising in this case [27,28]. The optical properties of photosynthetic pigments such as chlorophylls, bacteriochlorophylls, and carotenoids, as well as the pigment–protein complexes containing them, are the focus of intensive theoretical research [27,29,30]. The basic concept of semi-classical calculations is the spectral density function, which carries information about the vibronic structure of the molecule and about the intensity of interaction of different vibrational modes with electronic excitation [28]. However, modeling with semi-classical theories has one drawback, which is the need to adjust microparameters that cannot be measured directly, and their values can only be estimated be fitting experimental spectra. A successful solution to this problem was the application of a multiparametric optimization algorithm—differential evolution [31,32]. The development of quantum models of the $\left|\left.S_{0}\right\rangle \right.\to \left|\left.S_{2}\right\rangle \right.$ electronic transition of carotenoids and the $\left|\left.S_{0}\right\rangle \right.\to \left|\left.Q_{y}\right\rangle \right.$ transition of chlorophylls and bacteriochlorophylls, as well as the fitting of experimental spectra, were implemented by using differential evolution [33,34,35].

It is clear that even semi-classical calculations are time consuming, and it can be difficult to use them directly with spectrometric instruments. One of the possible simplifications is to apply an approach when the optical spectra of dye molecules are approximated by a set of bell-shaped Gaussian curves [12,14,23,36,37,38]. It must be stressed that from the point of view of quantum theory, such presentation can be considered as a rough approximation. Thus, the 0-0 electronic transition band of Chl (BChl) in solution at room temperature in the frequency domain is described by a rather narrow Gaussian function of large amplitude. The phonon wing components give a broad band of smaller amplitude lying towards higher frequencies (higher energy). Doppler broadening and other interactions lead to some deviation of the shape of the electronic transition band from Gaussian. Moreover, this shape deviation can easily be taken into account by adding one more or, in extreme cases, two Gaussian bands. Yet, more Gaussian bands are required to fit the electronic transition of carotenoids than in the case of electronic transition of chlorophylls. Thus, we are faced with the main problem of the decomposition into Gaussian bands: the approximation of the spectrum by Gaussian curves is ambiguous and the success of the decomposition depends on the correct initial choice of the position, width, and amplitude of each Gaussian curve [19].

**Figure 1:**
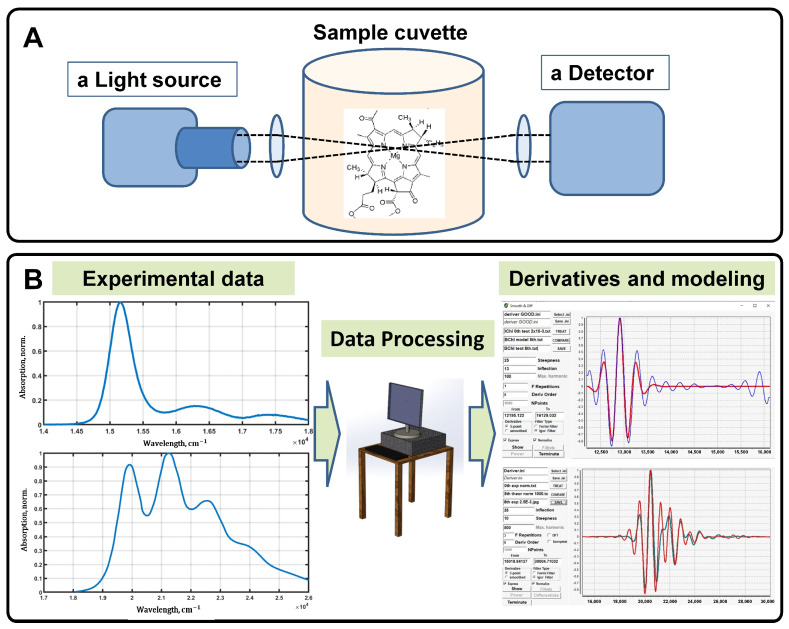
General scheme of a spectrophotometric setup, data processing, and modeling. The light source, sample cuvette, and detector, which are the essential elements of any spectrophotometer, allow for measuring the optical properties of pigment molecules in solvents as a function of wavelength (**A**). The measured spectra are subjected to the proper processing, in particular, signal smoothing is performed, which is necessary for its further analysis and modeling (**B**).

Thus, the main goal of our work is to demonstrate that Gaussian decomposition of experimental absorption spectra of photosynthetic pigments can be used a suitable approximation of the shape of fitted spectra (for further calculation of high order undistorted derivatives) instead of simulating the linear optical response within the framework of semi-classical quantum theory. Moreover, taking into account the computational cost of data processing, Gaussian decomposition is much faster than modeling the absorption profile using semi-classical quantum theories; it can then be argued that this method of data approximation can be used directly in spectroscopic devices (remote sensing) for on-the-fly data processing (Figure 1).

## 2. Materials and Methods

### 2.1. Absorption Spectra of Photosynthetic Pigments

**Figure 2:**
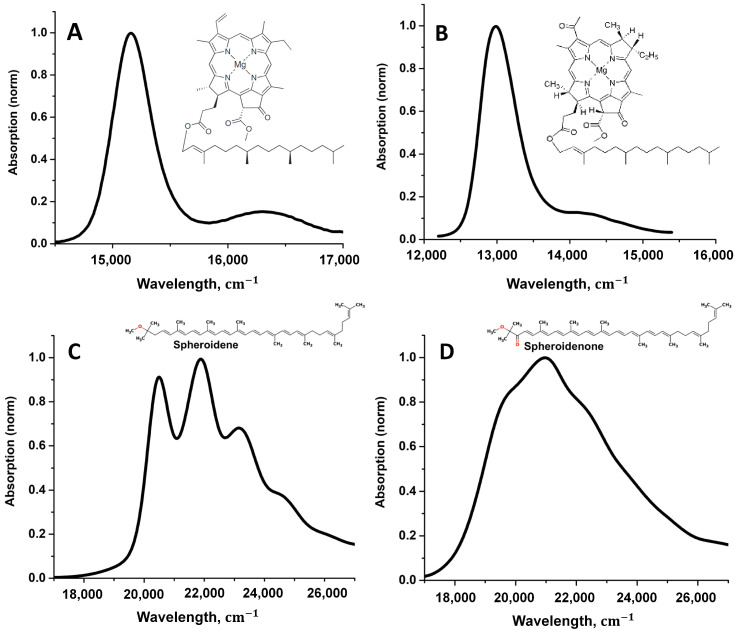
Experimentally measured absorption spectra of Chl (**A**), BChl (**B**), spheroidene (**C**), and speroidenone (**D**) in solvents at room temperature.

To perform the optical properties simulations, absorption spectra of Chl, BChl, spheroidene, and spheroidenone were taken from the published studies [35,39]. All spectra were measured at room temperature; Chl and BChl were diluted in dimethyl ether and taken in a range from 300 to 800 nm with a step of 1 nm, while spheroidene and spheroidenone were diluted in a 7/2 (*v*/*v*) acetone/methanol mixture and in a range from 450 to 550 nm (Figure 2).

### 2.2. Multimode Brownian Oscillators Model

**Figure 3:**
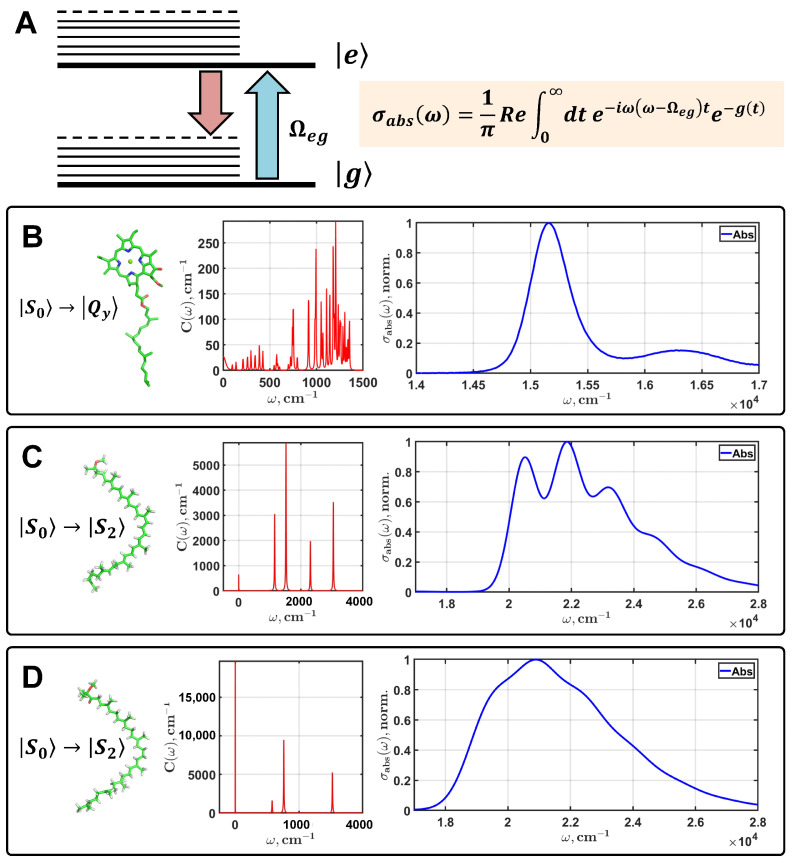
Theoretical modeling of the linear optical response of photosynthetic pigments within the framework of multimode Brownian oscillator theory. A diagram of the electronic transition from the ground $\left|\left.g\right\rangle \right.$ to the excited $\left|\left.e\right\rangle \right.$ state is shown in plot (**A**). Thick lines are the electronic states; thin lines are the manifold of vibronic states of a pigment interacting with the electronic states. $\Omega_{ge}$ is the electronic energy gap. $\sigma_{abs}\left(\omega \right)$ is an integral expression for the absorption spectrum depending on the lineshape function $g\left(t\right)$. The simulated spectra of the $\left|\left.S_{0}\right\rangle \right.\to \left|\left.Q_{y}\right\rangle \right.$ transition of Chl (**B**) and the $\left|\left.S_{0}\right\rangle \right.\to \left|\left.S_{2}\right\rangle \right.$ transition of shperoidene (**C**) and spheroidenone (**D**) are presented. Red lines are the spectral densities of pigments; the linear absorption spectra are blue.

The bands of different intensities and widths observed in the absorption spectrum of an organic pigment in the visible wavelength range are the result of the transition from one electronic state to another. Assuming that the molecule can be in the ground $\left|\left.g\right\rangle \right.$ and excited $\left|\left.e\right\rangle \right.$ electronic states, it is then possible to simulate the absorption spectrum profile using a semi-classical theory called the multimode Brownian oscillator model (Figure 3A).

Within the framework of the applied theory, the number of vibronic modes associated with an electronic state is determined by the spectral density function (Figure 3B–D). Moreover, it is assumed that these vibrations of the molecular skeleton interact with the bath modes, which allows us to take into account the influence of the local surroundings. Each vibronic mode is represented by three parameters, namely the frequency $\omega_{j}$, the relaxation rate $\gamma_{j}$, and the Huang–Rhys factor $S_{j}$. Depending on the ratio between $\omega_{j}$ and $\gamma_{j}$, different types of nuclear motions can be represented. $S_{j}$ is the effective interaction energy of *j*th mode with the electronic state. The expression for the spectral density function is

(1) $$C^{\prime \prime }\left(\omega \right)=\sum_{j}\frac{2S_{j}\omega_{j}^{3}\omega \gamma_{j}}{{\left(\omega_{j}^{2}-\omega^{2}\right)}^{2}+\omega^{2}\gamma_{j}^{2}},$$

$C^{\prime \prime }\left(\omega \right)$ is a real function that is used to calculate the complex correlation function of the electronic energy gap between $\left|\left.g\right\rangle \right.$ and $\left|\left.e\right\rangle \right.$ states [28]. The temperature dependent correlation function is then written as

(2) $$g\left(t\right)=\frac{1}{2\pi }\int_{-\infty }^{\infty }d\omega \frac{1-\cos\omega t}{\omega^{2}}\coth\left(\beta \hbar \omega /2\right)C^{\prime \prime }\left(\omega \right)-\frac{i}{2\pi }\int_{-\infty }^{\infty }d\omega \frac{\sin\left(\omega t\right)-\omega t}{\omega^{2}}C^{\prime \prime }\left(\omega \right),$$

This function is also called the line-shape function and is used in the expression for the absorption spectrum lineshape:

(3) $$\sigma_{abs}\left(\omega \right)=\frac{1}{\pi }Re\;\int_{0}^{\infty }dt\;e^{i\left(\omega -\Omega_{eg}\right)t}e^{-g\left(t\right)}e^{-\frac{1}{2}{\left(\Delta t\right)}^{2}},$$

Here, $\Omega_{eg}$ is the 0-0 frequency of the electronic transition $\left|\left.g\right\rangle \right.\to \left|\left.e\right\rangle \right.$. $\Delta =FWHM/2\sqrt{2\cdot ln2}$ is the inhomogeneous broadening parameter. The inhomogeneous broadening of absorption is an effect due to the influence of the immediate surroundings on the electronic transition of the pigment (the effect of the solvent). FWHM is the full width at the half maximum of Gaussian distribution.

Thus, to simulate a realistic absorption spectrum according to (3), we need to optimize the following parameters of the quantum model: $\Omega_{eg}$, FWHM, and $\left\{\omega_{j},S_{j},\gamma_{j}\right\}$, where $j\in \left[1,\;\ldots ,n\right]$, n is the number of vibronic modes specific for a pigment.

### 2.3. The Eighth Derivative Calculation Procedure

Original software was developed to process the experimental spectra. It allows us to upload and download data files, visualize the spectra, as well as smooth the original data and calculate derivatives of any order. The procedure of gaining high-order derivatives of the pigment absorption spectrum using Gaussian decomposition involves several steps. Considering the long-wavelength absorption band of chlorophyll a as an example, let us explain the series of operations that was used in our calculations. Initially, the Chl spectrum was measured in diethyl ether at room temperature in a range from 300 to 800 nm with a step of 1 nm. Only the part of the spectrum from 600 to 800 nm (the so-called Qy band) was taken for further processing (Figure 2A). This spectral region is used for Gaussian decomposition. The main electronic peak of the Qy band is shifted to the center of the figure to avoid edge effects during differentiation. 

**Figure 4:**
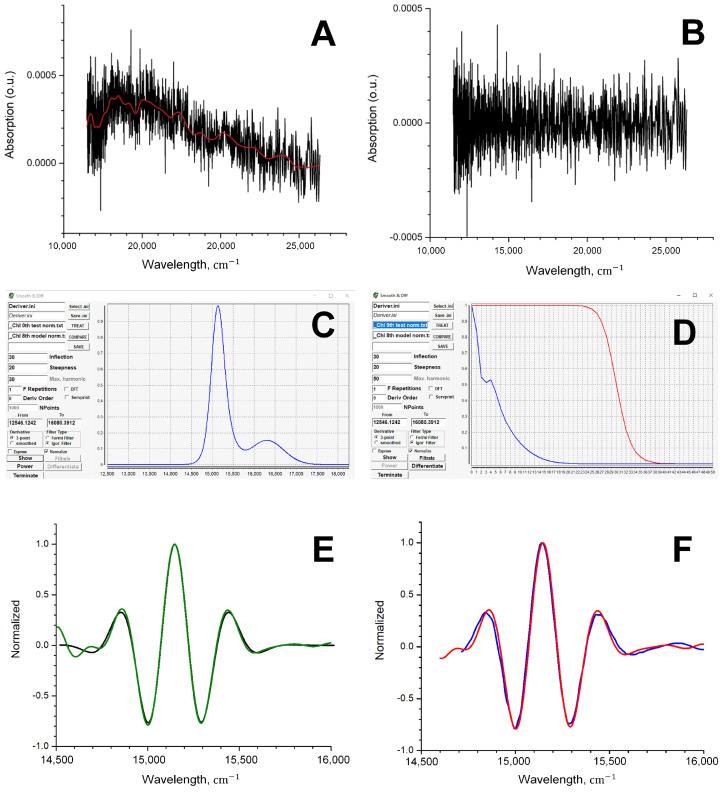
The noise line obtained on the spectrophotometer when registering without a cuvette in the sample compartment; the red curve is a smoothed noise line (**A**). The straightened noise obtained from the noise line by subtracting the smoothed noise line (**B**). Search for optimal FFT filter parameters using Chl as an example. The workspace of Deriver602green program: (**C**)—processed spectrum, (**D**)—the frequency representation of the spectrum (blue) and the filter curve (red). (**E**) Normalized eighth derivatives of the test (green) and model (black) Chl spectra. (**F**) Normalized eighth derivatives of the experimental (red) and theoretical (blue) Chl spectra.

The input parameters of the procedure of approximation of absorption spectra by Gaussians are the number of Gaussians, their intensities, the width at half maximum, and the frequency shift. In general, the fitting can be optimized by using any multiparametric optimization [19], but specifically for Chl and carotenoid spectra, acceptable results can be obtained without optimization. It should be emphasized that the approximated spectrum is a smooth curve, for which it is easy to obtain the derivative of any order. However, the experimental absorption spectrum is an array of values measured to an accuracy ±0.0001 (if the maximum of intensities is normalized to 1.0) or worse. When recorded on a spectrophotometer, this measurement error gives a “noise track”, the amplitude of which can vary due to the different sensitivity of the instrument at different wavelengths (Figure 4A,B).

To simulate the effect of real measured data, a noise track taken on a spectrophotometer with an empty cuvette was used. This straightened noise array is added to the model spectrum and the test spectrum is obtained (Figure 4A). The test spectrum and the experimental spectrum contain approximately the same noise component. Therefore, the test spectrum can be used to find the parameters of the best filter (Figure 4C,D). By applying filtering (smoothing) and differentiation of the test spectrum, you can obtain, for example, its eighth derivative. However, the exact form of this eighth derivative is already known (this is the eighth derivative of the model spectrum). Comparing the eighth derivatives of the test spectrum and the model spectrum, one can choose the filter parameters that give the best match for these derivatives. This filter will be called the optimal filter. The use of this optimal filter to obtain the eighth derivative of the experimental Chl spectrum should ensure good agreement between the eighth derivatives of the experimental and theoretical Chl spectra (Figure 4E,F).

## 3. Results

It is well known that the shape of absorption spectra of photosynthetic pigments is determined by the intensity of the nuclear motions of molecular skeleton [28,37]. Applying the multimode Brownian oscillator model, these types of motions can be taken into account by evaluating the spectral density function [33,34]. In this study we will distinguish between spectra calculated theoretically using spectral density and spectra obtained using Gaussian decomposition.

Parameters of the multimode Brownian oscillator model for simulation of the theoretical absorption spectra of Chl (Figure 3B) and BChl were taken from our previous studies [35,40]. Carotenoid spectra were simulated especially for this study (Figure 3C,D). Parameters of the spectral density, the electronic energy gap, and the full width at half maximum are listed in Table A1 in Appendix A. The time and frequency scales of simulated spectra contain $2^{12}=4096$ points. This is due to the use of the fast Fourier transform to process the spectra, which requires $2^{n}$ point arrays. All the spectra were saved with an accuracy of 16 digits. Numerical differentiation of such simulated data makes it possible to obtain derivatives up to the eighth order without preliminary smoothing, namely without distortion. It has to be stressed that the eighth derivative of the spectrum of any dye is the natural limit for a standard personal computer when using double precision. When calculating derivatives of a higher order, there are problems related to the representation of numbers in the RAM of the computer.

**Figure 5:**
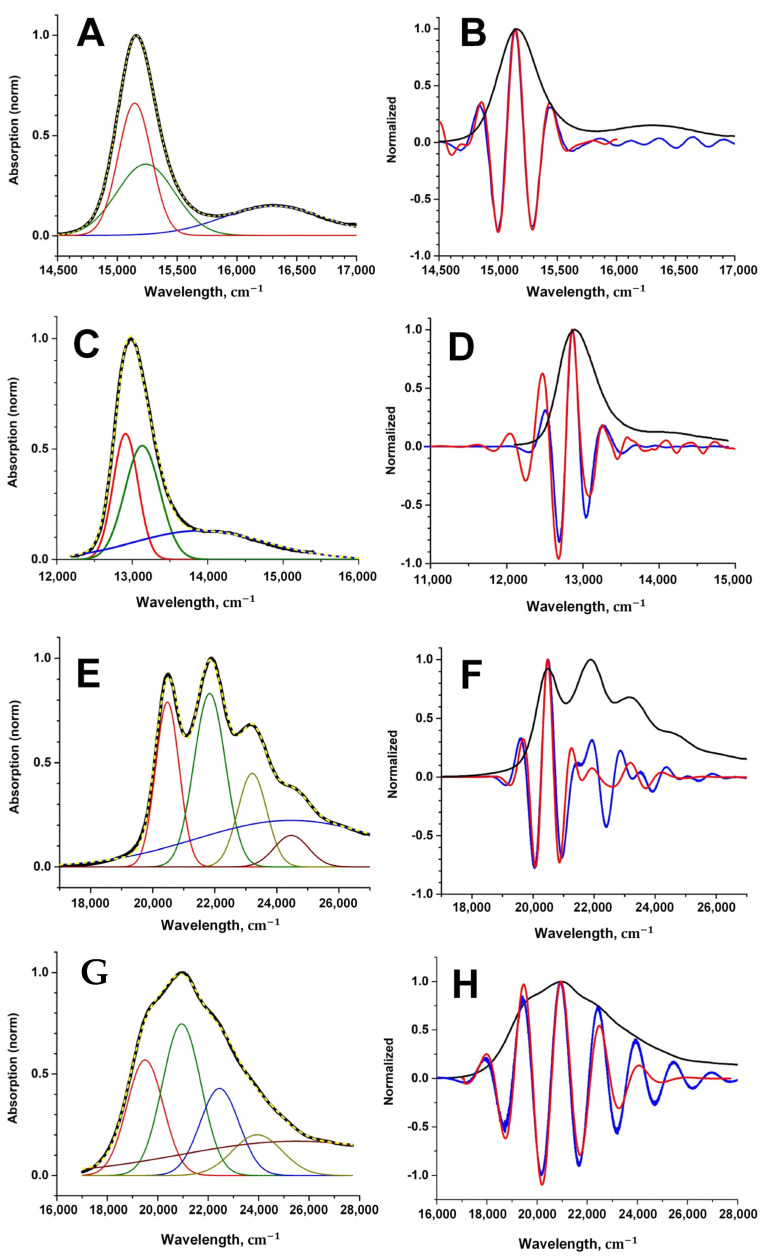
Experimental absorption spectra of Chl (**A**), BChl (**C**), (black curves) and its decomposition into three Gaussian bands (red, green, and blue), as well as experimental spectra of spheroidene (**E**) and spheroidenone (**G**) and its decomposition into five Gaussian bands. The resulting spectra, which are the sum of Gaussians, are shown by the yellow dashed lines. Normalized eighth derivatives of the pigment experimental spectra (red curves) and those of theoretical spectra (blue curves) are shown on the graphs (**B**,**D**,**F**,**H**).

Spectra obtained by Gaussian decomposition are much easier to simulate. Using the procedures described in the previous section and applying our software, we can approximate the experimental spectra with a high degree of fidelity (Figure 5A,C,E,J). The key point is to find the optimal filter parameters. The use of test spectra for smoothing allowed us to adjust the filter finely and we obtained the smallest discrepancies between the spectrum of an ideal higher derivative (for example, of the eighth order) and the corresponding model derivative.

To check that the proposed optimal filter parameter selection technique works, we applied it to obtain the eighth derivatives of the experimental spectra of four photosynthetic pigments for which the theoretical spectra were calculated (Chl, BChl, spheroidene, and spheroidenone). The results are shown in Figure 5. It can be seen that the proposed technique makes it possible to obtain high derivatives of the experimental spectrum, which are quite close to the theoretical ones.

## 4. Discussion

Application of the Gaussian decomposition has a long tradition, particularly in astronomy [19,41,42]. The optical data obtained by astronomers are essentially different from the spectroscopy of biological pigments and proteins. Astronomers measure and analyze the intensities of atomic transitions, whereas the object of study for biophysicists is the electronic transitions of multi-atomic organic molecules, complicated by the influence of the immediate environment (solvents or proteins). Electronic transitions in atoms and small inorganic molecules in space mostly have a Gaussian shape, therefore decomposition into Gaussians, in addition to a trivial smoothing of spectral curves and noise removal, allows astronomers to extract from the absorption and emission spectra parameters of physical processes (for instance, the Doppler shift of frequencies, the full width at half maximum) occurring in the interstellar space. The computational implementation of modern advances in artificial intelligence enables efficient automation of astronomical data processing [13,19,43].

Unlike the spectra of astronomical objects, the spectra of biological pigments are much more difficult to interpret. In general, the shape of their absorption profile is not Gaussian and is determined by the integral expression (3). This expression can turn to the Gaussian function only at a certain relation between the parameters of the theory, which are in fact never fulfilled for organic pigments at room temperature [28,30,37]. Experimental spectra of photosynthetic pigments are shown in Figure 2. It should be noted that the Gaussian decomposition in the study of organic and photosynthetic pigments has its own history [44,45,46,47]. However, in spite of the achieved success, one cannot but note the fact that the parameters of Gaussians obtained after decomposition have no physical meaning and do not reflect real physical processes occurring in pigments when absorbing light quanta. On the other hand, the high derivatives of the Chl and BChl spectra in these studies are characterized by a large number of narrow peaks, which were attributed to the absorption bands of certain transitions, which in fact can be interpret as insufficient filtering (smoothing) of the original spectra. Interestingly, in addition to decomposition into Gaussians or Lorentzians, the Padé transform was used to estimate the shape of the derivative spectrum, which, however, also excludes the possibility of physical interpretation of the obtained data [48].

The absorption spectra of Chl and BChl in the range of 12,000–17,000 cm^−1^ (830–590 nm) and carotenoids in the range of 18,000–26,000 cm^−1^ (560–380 nm) were not arbitrary chosen. It is in these ranges that the absorption bands of strictly defined electronic transitions are found. Thus, for this spectral region it is allowed to use the multimode Brownian oscillator model, which will reproduce the true absorption profile with high accuracy. With the help of the differential evolution algorithm, we have fitted the given spectra finely [35], however, from a practical point of view, if we are talking about automation of the processes of analysis and registration of optical data, carrying out such modelling will not allow us to analyze the spectra on the spot. Moreover, if we consider the problem more generally, it is obvious that the set of vibronic modes of any organic pigment cannot be known exactly. This circumstance fundamentally complicates the application of the multimode Brownian oscillator model and the use of this theory becomes irrational if the purpose of modeling is to obtain high-order derivatives. It is worth noting that the application of semi-classical theories to on-the-fly modeling of optical properties has been discussed before [15,16,18].

Consequently, to calculate higher order derivatives, we replace the approximation of the experimental spectrum based on a physical model (discussed above) with the approximation of the experimental spectrum by the sum of Gaussians. It is clear that such an envelope is an analytic curve for which it is easy to obtain accurate high derivatives. We call the envelope the model curve, and its derivatives, the model derivatives. By adding the noise track to the model spectrum, we obtain a test spectrum that approximates the experimental one in its shape and noise characteristics.

Smoothing (filtering) the test spectrum with the selected filter, we obtain its eighth derivative and compare it with the model eighth derivative. The process continues until it is possible to obtain the best match of these two derivatives and thereby find the parameters of the optimal filter. Since it is assumed that the noise characteristics of the test spectrum do not differ from the noise characteristics of the experimental one, the use of the optimal filter for processing the experimental spectrum should allow one to obtain its “true” eighth derivative. For the four compounds that are considered in this work, it is possible to check whether their eighth derivatives obtained using the proposed method are really “true”. As can be seen from the comparison of the derivatives obtained by smoothing/differentiating the experimental spectra and the theoretical derivatives (Figure 5), the agreement is quite good.

## 5. Conclusions

It was shown that the noisy experimental spectra of photosynthetic pigments can be processed by Gaussian decomposition in order to obtain the undistorted high-order derivatives. The spectra of the studied pigments (chlorophyll, bacteriochlorophyll, and carotenoids) were chosen in a way that their frequency range allowed the use of a semi-classical theory called the multimode Brownian oscillator model to simulate the shape of the absorption spectrum. The calculated true theoretical spectra were used as reference spectra to generate higher derivatives, in particularly the eighth one. At the same time, the absorption spectra were modeled using Gaussian decomposition: three Gaussians were sufficient to obtain the envelope curve of the spectrum for Chl and BChl, and five Gaussians were needed in the case of carotenoids. Overlaying a noise track on the model spectra allowed us to synthesize the test experimental data roughly, as it would be read from a spectrophotometer. By comparing the model derivatives of the eighth order and those of the test spectra, the optimal settings of the smoothing filter were found, where the spectra of the high derivatives match perfectly.

Thus, it was demonstrated that Gaussian decomposition in modeling the optical response of biological pigments allows us to obtain undistorted high-order derivatives with good precision. Considering that the computational costs of Gaussian decomposition is much less than optimization of spectra modeling by semi-classical quantum theory, this method can be used in on-the-fly spectral data analysis with minimal computational costs. It is also worth noting that in the future it is reasonable to consider the possibility of optimizing the Gaussian decomposition with the help of differential evolution. The algorithm of multiparametric optimization has already shown high efficiency in quantum calculations of the optical response of photosynthetic pigments [33,34,35].

## Data Availability

Not applicable.

## Appendix A

**Table A1:** Parameters of the multimode Brownian oscillator model for simulation of spheroidene (Figure 3C) and spheroidenone (Figure 3D) absorption spectra. $\nu_{1}=1524\;{cm}^{-1}$, $\nu_{2}=1158\;{cm}^{-1}$, $\nu_{3}=1006\;{cm}^{-1}$, $\nu_{4}=965\;{cm}^{-1}$ are the characteristic vibronic modes of carotenoids; 2$\nu_{1}$ and 2$\nu_{2}$ are the overtones of the corresponding frequencies; $\nu_{1}+\nu_{2}$ is sum of the frequencies; $S_{j}$ are Huang-Rhys factors, where $j=\left\{\nu_{1},\nu_{2},\nu_{3},\nu_{4},\;2\nu_{1},2\nu_{2},\nu_{1}+\nu_{2}\right\}$; $\Omega_{eg}$ is the electronic energy gap; ${FWHM}_{\Omega }$ is the full width at half maximum.

	$\Omega_{eg}$	${FWHM}_{\Omega }$	$\omega_{low}$	$S_{low}$	$\gamma_{low}$	$S_{\nu_{1}}$	$S_{\nu_{2}}$	$S_{\nu_{3}}$	$S_{\nu_{4}}$	$2\nu_{2}$	$2\nu_{1}$	$\nu_{1}+\nu_{2}$
Spheroidene	22,631.0	1007.6	4.9	3.0	42.7	0.63	0.57	7.3 × 10^−3^	1.5 × 10^−3^	9.3 × 10^−2^	6.9 × 10^−8^	9.7 × 10^−2^
Spheroidenone	21,785.8	1668.3	1.0	3.2 × 10^−3^	450.4	1.02	0.30	3.2 × 10^−11^	2.5 × 10^−11^	1.1 × 10^−11^	1.2 × 10^−11^	0.15
